# Interaction between *cyclooxygenase-2* gene polymorphism and dietary n-6 polyunsaturated fatty acids on colon cancer risk: The Singapore Chinese Health Study

**DOI:** 10.1038/sj.bjc.6601797

**Published:** 2004-04-13

**Authors:** W-P Koh, J-M Yuan, D van den Berg, H-P Lee, M C Yu

**Affiliations:** 1 Department of Community, Occupational and Family Medicine, National University of Singapore, MD3, 16 Medical Drive, Singapore 117597, Singapore; 2 USC/Norris Comprehensive Cancer Center, Keck School of Medicine, University of Southern California, Los Angeles, CA, USA

**Keywords:** cyclooxygenase-2, n-6 polyunsaturated fatty acids, colon cancer, Chinese

## Abstract

This case–control study of 310 colorectal cancer cases and 1177 controls in a nested prospective, population-based cohort of Singapore Chinese subjects found a statistically significant association between the cyclooxygenase (COX)-2 −*765G*>*C* gene polymorphism and colon cancer risk among high consumers of dietary n-6 polyunsaturated fatty acids (odds ratio=2.38, 95% confidence interval=1.23–4.59).

Diet is believed to be the single most important contributor to colonic carcinogenesis (Tomatis *et al*, 1990). Experimental data have shown that saturated fatty acids (SFAs) and n-6 polyunsaturated fatty acids (PUFAs) have tumour-enhancing properties in the colon (Reddy and Maeura, 1984; Zhao *et al*, 1991, Woutersen *et al*, 1999). Epidemiological data suggest that increased consumption of all meat or red meat, which contains high levels of SFAs, is strongly associated with colorectal cancer (Giovannucci and Goldin, 1997; Sandhu *et al*, 2001), but there is only limited evidence on the role of dietary n-6 PUFAs (Zock and Katan, 1998; Flood *et al*, 2003).

The putative mechanism through which dietary n-6 PUFAs may enhance colonic carcinogenesis is the increased formation of prostaglandins, with the rate-limiting and committal step being mediated by the cyclooxygenase (COX)-2 enzyme (Dubois *et al*, 1998). Prostaglandins possess a wide spectrum of procarcinogenic properties (Handler *et al*, 1990; Cowlen and Eling, 1993; Coffey *et al*, 1997; Dermott *et al*, 1999). We therefore hypothesised that functional *COX-2* gene polymorphisms may impact on the conversion of n-6 PUFAs into prostaglandins, with consequent change in level of cancer risk. A single nucleotide polymorphism (−*765G*>*C)* in the promoter region of the *COX-2* gene was recently described (Papafili *et al*, 2002). We therefore investigated whether this *COX-2* gene polymorphism was related to colorectal cancer risk within a population-based, prospective cohort of middle-aged and older Chinese men and women in Singapore.

## MATERIALS AND METHODS

### Study subjects

The study design and subject recruitment of the Singapore Chinese Health Study have been described (Hankin *et al*, 2001). Briefly, 63 257 Chinese women and men aged 45–74 years belonging to the Hokkien or Cantonese dialect group were enrolled in the study between April 1993 and December 1998. At recruitment, information on lifestyle factors and usual diet over the last year was obtained through in-person interviews. The dietary component of the questionnaire was validated through a series of 24-h food recalls (Hankin *et al*, 2001). Respondents were asked to choose from predefined frequency and portion size categories for each of the 165 listed food/beverage items that he/she consumed during the past 12 months. We used the Singapore Food Composition Table to estimate average daily intake of 96 nutrient and non-nutrient compounds for each study subject (Hankin *et al*, 2001). The Institutional Review Boards at the University of Southern California and the National University of Singapore had approved this study.

We identified incident colorectal cancer cases through the population-based cancer registry in Singapore (Chia *et al*, 2000). As of 30 April 2002, 592 colorectal cancer cases had occurred among cohort participants. All cases (including one carcinoid tumour and two *in situ* cancers) were histologically confirmed except three (ascertained by death records and clinical evidence). Details of the biospecimen collection, processing and storage procedures have been described (Koh *et al*, 2003). Briefly, we attempted to collect blood and single-void urine specimens from a random 3% sample of cohort enrollees. If the subject refused to donate blood, he/she was asked to donate buccal cells. We collected blood/buccal cell samples from 1194 subjects during April 1994–July 1999. Of these subjects, 13 developed colorectal cancer by 30 April 2002, and the remaining 1181 subjects constituted the referent group for the present study. We also attempted to collect blood/buccal cell and urine samples from all incident colorectal cancer cases. Of the 592 colorectal cancer cases, 312 (53%) donated blood/buccal cell samples.

### COX-2 genotyping

Genomic DNA was extracted from buffy coats (228 cases and 895 controls) and buccal cell samples (84 cases and 286 controls) using a QIAamp 96 DNA Blood Kit (Qiagen, Valencia, CA, USA). A TaqMan assay for the −*765G*>*C COX-2* polymorphism was developed using a TaqMan PCR Core Reagent kit (Applied Biosystems Inc., Foster City, CA, USA). The oligonucleotide primers for amplification of the polymorphic region of *COX-2* were GC093 for (5′-CATTAACTATTTACAGGGTAACTGCTTAGG-3′) and GC093rev (5′-CCCCCTCCTTGTTTCTTGGA-3′). In addition, the fluorogenic oligonucleotide probes (TaqMan MGB Probes; ABI) used to detect each of the alleles were GC093F (5′-CTTTCCCGCCTCTCT-3′) labelled with 6-FAM to detect the *G* allele and GC093V (5′-CTTTCCCCCCTCTCT-3′) labelled with VIC to detect the *C* allele. Experimental samples were compared to 12 controls to identify the three genotypes at each locus (*GG, GC, CC*). All samples were processed without knowledge of their case/control status. Any samples that were outside the parameters defined by the controls were identified as noninformative and were retested. Four controls and two cases had noninformative *COX-2* genotypes and were excluded from the present analysis.

### Statistical analysis

Data were analysed by standard methods for unmatched case–control studies (Breslow and Day, 1980). Unconditional logistic regression models were used to examine the associations between *COX-2* genotypes and risk of colorectal cancer, and their possible modification by n-6 PUFA intake. The associations were measured by odds ratios (ORs) and their corresponding 95% confidence intervals (CIs) and *P*-values (two-sided). Limited by the very low frequency of the *CC* genotype (0.003), the *GC* and *CC* genotypes were combined when compared with the *GG* genotype. All ORs were adjusted for age (year) at recruitment, year of recruitment, gender, dialect group (Cantonese, Hokkien), level of education (no formal schooling, primary school, secondary school and higher), body mass index (<20, 20 to <24, 24 to <28, 28+ kg m^−2^), smoking status (never, exsmoker, current smoker), frequency of alcohol consumption (nondrinker, monthly drinker, weekly drinker, daily drinker), and familial history of colorectal cancer (yes, no).

## RESULTS

Of the 592 incident colorectal cancer cases, 282 were excluded from the present analysis due to unavailable blood/buccal cell samples (*n*=280) or noninformative *COX-2* genotype (*n*=2). Cases included in the present study (*n*=310) were comparable to those excluded in terms of age (mean: 65.4 *vs* 66.1 years), but slightly different in gender (57 *vs* 49% male), dialect group (45 *vs* 37% Cantonese) and level of education (69 *vs* 60% attaining primary school education or higher).

In total, 180 (58%) cases had colon cancer, and the remaining cases had either rectal or rectosigmoid cancers. Table 1

**Table 1:** Selected characteristics of colorectal cancer cases and controls, the Singapore Chinese Health Study

**Characteristics**	**Controls (*n*=1177)**	**Cases (*n*=310)**	***P*-value^a^**
Mean age±s.d.^b^ (years)	56.5±8.1	61.3±7.5	<0.001
			
	Number (%)		
*Sex*			
Males	509 (43.2)	178 (57.4)	<0.001
Females	668 (56.8)	132 (42.6)	
			
*Dialect group*			
Cantonese	571 (48.5)	138 (44.5)	0.23
Hokkien	606 (51.5)	172 (55.5)	
			
*Level of education*			
No formal schooling	318 (27.0)	95 (30.6)	0.01
Primary school	504 (42.8)	151 (48.7)	
Secondary school	288 (24.5)	54 (17.4)	
A level/university	67 (5.7)	10 (3.2)	
			
*Body mass index* (*kg* *m*^−*2*^)			
⩽20	187 (15.9)	46 (14.8)	0.04
20−<24	659 (56.0)	151 (48.7)	
24−<28	267 (22.7)	92 (29.7)	
28+	64 (5.4)	21 (6.8)	
			
*Cigarette smoking*			
Never smokers	853 (72.5)	184 (59.4)	<0.001
Former smokers	131 (11.1)	52 (16.8)	
Current smokers	193 (16.4)	74 (23.9)	
			
*Frequency of alcohol consumption*			
Nondrinkers	964 (81.9)	243 (78.4)	0.14
Monthly	85 (7.2)	21 (6.8)	
Weekly	93 (7.9)	29 (9.4)	
Daily	35 (3.0)	17 (5.5)	
			
*Familial history of colorectal cancer*			
No	1149 (97.6)	297 (95.8)	0.12
Yes^c^	28 (2.4)	13 (4.2)	
			
*Median (5th percentile, 95th percentile)*			
Total calories (kcal day^−1^)	1483.5 (829.9, 2477.5)	1494.0 (819.3, 2589.2)	0.54
Total fat (g day^−1^)	40.5 (19.6, 81.7)	39.8 (19.0, 77.1)	0.66
SFAs (g day^−1^)	14.0 (5.9, 30.0)	13.7 (5.8, 29.2)	0.62
MUFAs (g day^−1^)	13.6 (6.3, 28.0)	13.4 (6.1, 26.5)	0.88
PUFAs (g day^−1^)	8.1 (3.3, 18.6)	7.9 (3.4, 16.5)	0.74
N-3 PUFAs (g day^−1^)	0.8 (0.4, 1.7)	0.8 (0.4, 1.7)	0.94
N-6 PUFAs (g day^−1^)	7.2 (2.9, 16.8)	7.0 (3.0, 14.9)	0.75
Fiber (g day^−1^)	12.2 (5.0, 23.1)	11.9 (4.3, 22.5)	0.83
Calcium (mg day^−1^)	373.5 (156.7, 853.5)	363.4 (158.8, 773.6)	0.56
Folate (*μ*g day^−1^)	146.0 (64.1, 277.7)	147.1 (57.9, 286.9)	0.65

a Two-sided *P*-values derived from *t*-test (for age), *χ*^2^ test (for categorical variables) or Wilcoxon rank sum test (for nutrient intakes).

b Mean age at recruitment into the cohort, s.d.=standard deviation.

c Any of first-degree relatives had colorectal cancer.

shows the distributions of selected characteristics of colorectal cases and controls. Cases were older, less educated, more obese and more likely to smoke cigarettes than controls. Intakes of total calories, total fat, SFAs, monounsaturated fatty acids (MUFAs), PUFAs, n-3 PUFAs, n-6 PUFAs, fibre, calcium or folate were comparable between cases and controls.

Among control subjects, the *G* and *C* allele frequencies of the *COX-2* genotype were 0.952 and 0.048, respectively, and the *GG*, *GC* and *CC* genotype frequencies were 0.907, 0.090 and 0.003, respectively. These genotypic distributions were in Hardy–Weinberg equilibrium (*P*=0.43). Overall, there was no association between colorectal cancer risk and *COX-2* −*765G*>*C* genotype or n-6 PUFA intake (Table 2

**Table 2:** *COX-2* −*765G*>*C* genotype and dietary intake of n-6 PUFAs in relation to risk of colorectal cancer, the Singapore Chinese Health Study

		**Colorectal cancer**		**Colon cancer**		**Rectal cancer**	
	**Controls**	align="center"**Cases**	**OR (95% CI)^a^**	**Cases**	**OR (95% CI)^a^**	**Cases**	**OR (95% CI)^a^**
*COX-2 genotype*							
GG	1067	273	1.00	155	1.00	118	1.00
GC/CC	110	37	1.18 (0.77–1.79)	25	1.50 (0.92–2.47)	12	0.87 (0.45–1.66)
							
*Dietary n-6 PUFAs in quartile*^b^							
1st (low)	260	70	1.00	42	1.00	28	1.00
2nd	297	83	1.08 (0.72–1.60)	48	1.15 (0.70–1.87)	35	0.97 (0.55–1.71)
3rd	312	72	1.00 (0.65–1.53)	45	1.09 (0.64–1.83)	27	0.82 (0.44–1.54)
4th (high)	308	85	1.04 (0.63–1.70)	45	1.04 (0.56–1.92)	40	0.99 (0.49–1.99)

a ORs were adjusted for age at recruitment, year of recruitment, gender, dialect group, level of education, body mass index, smoking status, frequency of alcohol consumption, and familial history of colorectal cancer; CI=confidence interval.

b In addition to all variables listed above, OR were adjusted for total energy intake.

). When subjects were stratified into high (above median) *vs* low (below median) intake levels of n-6 PUFAs, a borderline statistically significant association between genotype and risk was observed among high consumers of n-6 PUFAs (OR=1.65, 95% CI=0.95–2.87), which was mainly confined to colon cancer (OR=2.38, 95% CI=1.23–4.59) (Table 3

**Table 3:** *COX-2* −*765G*>*C* genotype in relation to risk of colorectal cancer stratified by level of dietary n-6 PUFAs, the Singapore Chinese Health Study

		**Colorectal cancer**		**Colon cancer**		**Rectal cancer**	
	**Controls**	**Cases**	**OR (95% CI)^a^**	**Cases**	**OR (95% CI)^a^**	**Cases**	**OR (95% CI)^a^**
*Low dietary n-6 PUFAs*^b^							
GG	508	139	1.00	81	1.00	58	1.00
GC/CC	49	14	0.82 (0.42–1.59)	9	0.95 (0.43–2.09)	5	0.68 (0.25–1.89)
							
*High dietary n-6 PUFAs*^b^							
GG	559	134	1.00	74	1.00	60	1.00
GC/CC	61	23	1.65 (0.95–2.87)	16	2.38 (1.23–4.59)	7	1.09 (0.46–2.59)

a ORs were adjusted for age at recruitment, year of recruitment, gender, dialect group, level of education, body mass index, smoking status, frequency of alcohol consumption, and familial history of colorectal cancer; CI=confidence interval.

b Defined as less than or equal to the median (‘low’) and greater than the median (‘high’) intake level (6.96 g day^−1^) of dietary n-6 PUFAs among all cohort members.

). There was no association between genotype and rectal cancer risk regardless of dietary n-6 PUFA intake levels. There was indication of an interaction effect between *COX-2* genotype and dietary n-6 PUFAs in colon cancer (*P*=0.07), which was absent in rectal cancer (*P*=0.51). The corresponding *P*-value for the gene–diet interaction effect in colorectal cancer combined was 0.10.

## DISCUSSION

In this cohort of Singapore Chinese, we reported a statistically significant effect of the *COX-2* −*765G*>*C* gene polymorphism on colon cancer risk among subjects with high intake of dietary n-6 PUFAs. Our data support the hypothesis that COX-2 exerts its effects on colon carcinogenesis through its influence on prostaglandin synthesis from n-6 PUFAs.

The current study has several strengths. (1) Our prospective study design precludes the possibility of recall bias. Furthermore, reliable dietary nutrient estimates including n-6 PUFAs were assessed using a validated food frequency questionnaire. (2) The nationwide cancer registry has been in place in Singapore since 1968 (Parkin *et al*, 2002), and migration out of Singapore has been negligible since inception of the study. This relatively complete ascertainment of cancer and death outcomes eliminates a concern for potential selection bias. (3) Study subjects originated from two contiguous regions in Southern China, resulting in a genetically homogeneous study population that facilitated the investigation of gene–disease associations. (4) Exposure information on other known/suspected risk factors for colorectal cancer was collected and accounted for in all analyses of gene–diet–cancer risk associations.

The chief limitation of our study is the lack of information on use of COX-2 inhibitors, which may bias the effect of *COX-2* genotype on risk. However, if use of COX-2 inhibitors were to exert a confounding effect on the observed COX-2 genotype/colon cancer association, our inability to control for such confounding is likely to lead to an underestimation, rather than an overestimation, of the risk associated with the putative high-activity genotype. This is because use of COX-2 inhibitors is likely to be more common among subjects with more severe symptoms of inflammation, possibly due to the possession of the high activity *COX-2* genotype. Another weakness of the present study is our relatively small number of cancer cases, which may result in less precise estimation of risk factor–disease associations.

The major n-6 PUFA in most diet is linoleic acid, the precursor of arachidonic acid. The latter is consequently converted to various prostaglandins, and COX is the crucial and rate-limiting enzyme for this conversion. There is compelling evidence that prostaglandins play important roles in colorectal carcinogenesis by enhancing cell proliferation and growth, promoting angiogenesis and inhibiting apoptosis (Cao and Prescott, 2002; Stoehlmacher and Lenz, 2003). *COX-2* gene expression and its mRNA and protein levels were markedly elevated in most human colorectal cancers relative to adjacent normal mucosa (Kargman *et al*, 1995; Sano *et al*, 1995). It is hypothesised that the COX-2-associated effect on colorectal carcinogenesis is due to the increased production of prostaglandins from dietary n-6 PUFAs (Eberhart and Dubois, 1995). In support of this hypothesis, high dietary n-6 PUFAs has been shown to promote colon tumorigenesis by upregulating COX-2 expression in animal studies (Singh *et al*, 1997).

The human *COX-2* gene is mapped to chromosome 1q25.2–q25.3 and spans about 8.3 kb pairs with 10 exons (Kosaka *et al*, 1994). Previous studies on the 5′ flanking region of the human *COX-2* gene show that this region contains a canonical TATA box as well as several putative factor elements that are critical in inducing *COX-2* gene transcription, such as Sp1, NF-*κ*B, GRE (glucocorticoid) and IRE (insulin) elements (Tazawa *et al*, 1994; Yang *et al*, 1997). The region from nucleotide −827 to −454 has been described as a negative region since deletion of this region led to increased luciferase activity in reporter expression studies. The −*765G*>*C* mutation lies within this region, and is also within one of the five putative Sp1 elements (Yang *et al*, 1997). At present, data on the functionality of the −*765G*>*C* polymorphism and the direction/magnitude of change in protein expression/activity between the *C* and *G* alleles are limited and mixed (Papafili *et al*, 2002; Schneider *et al*, 2003).

In summary, the present study provides the first epidemiological evidence for a possible cause-and-effect connection between the production of prostaglandins from n-6 PUFAs through the enzymatic activity of COX-2, and increased risk of tumour development in the colon. Our novel findings require confirmation in larger studies with varying levels of substrate intake and genotype frequency.
